# Voluminous Intrapericardial Lipoma Mimicking Pericardial Effusion

**DOI:** 10.1155/2020/6295634

**Published:** 2020-09-16

**Authors:** Fabíola Prado de Morais, Noah Romero Nakajima, Olívia Félix Marconi Andalécio, Pedro de Santana Prudente, Guilherme Emílio Ferreira, Andrea de Martino Luppi, Fernando Costa Mundim, Olga Maria Lima Aguiar, Juliana Salomão Daud Melo, Bruno de Carvalho Dornelas

**Affiliations:** ^1^Pathology Department, Universidade Federal de Uberlândia, Uberlândia, MG, Brazil; ^2^Medical School, Universidade Federal de Uberlândia, Uberlândia, MG, Brazil; ^3^Radiology Department, Universidade Federal de Uberlândia, Uberlândia, MG, Brazil

## Abstract

Lipomas are rare primary heart tumors and may involve the endocardium, myocardium, or pericardium. Signs and symptoms depend on the tumor location and size. The intrapericardial lipoma we report has massive dimensions and mimics a pericardial effusion. A 38-year-old male complained of dyspnea and precordial pain. On physical examination, heart sounds were diminished. The patient had received extensive medication for a clinically suspected pericardial effusion due to heart failure. A voluminous mass resembling fat within the pericardial sac was revealed by transesophageal echocardiography and a computed tomography scan. The tumor was removed successfully by a subxiphoid surgical approach. The diagnosis of a 635 gram intrapericardial lipoma was confirmed by pathological examination. After surgery, the patient recovered well and was completely asymptomatic at a follow-up at 90 days. No medications were being taken since. The diagnosis of a pericardial effusion should be secured by imaging exams to avoid unnecessary medications. Cardiac lipomas can be readily recognized by their typical features on radiologic imaging. The surgical pathology examination confirms the diagnosis and rules out malignancy criteria.

## 1. Introduction

Primary heart neoplasms are rare diseases, and their incidence rate is up to 0.4% in necropsy series [1, 2]. Approximately 75% of these lesions are benign tumors, such as myxomas, rhabdomyomas, lipomas, fibromas, and teratomas [3–5]. Lipomas represent approximately 10% of all primary cardiac tumors [4–9]. Additionally, about 25% of cardiac lipomas are intramyocardial, 25% are extracavitary with an epicardial origin, and 50% are intracavitary with a subendocardial origin [4, 10, 11]. They usually appear on the walls of the right atrium or left ventricle [4, 8, 11, 12]. Typically, lipomas are found in adults by the fifth and sixth decades of life. However, they can occur at any age. Cardiac lipoma frequency is about equal in both genders [5, 11, 13–15].

Signs and symptoms vary according to the size and localization of the tumor. Cardiac lipomas can cause thoracic pain, fatigue, dyspnea, syncope, arrhythmias, or even sudden death. Nevertheless, most cases are asymptomatic [4, 5, 8, 11, 16, 17]. Imaging exams are essential for guiding the diagnosis, such as echocardiography, tomography, or magnetic resonance. To exclude a malignant neoplastic process, the surgical pathology examination is necessary. Encapsulated lesions constituted of typical and mature adipocytes favor a benign neoplasm [5, 11]. In this article, we report the case of a young male patient with a large intrapericardial lipoma compressing the heart and mimicking a pericardial effusion.

## 2. Case Report

A 38-year-old male has complained of progressive dyspnea and sporadic precordial pain related to moderate effort for 5 months. Physical examination showed normal peripheral perfusion, and no edema was detected. On auscultation, no murmurs were heard; however, heart sounds were diminished. Clinically, a pericardial effusion due to heart failure was suspected and treated with pharmacologic drugs. The patient received digoxin, aspirin, furosemide, spironolactone, and carvedilol for 8 weeks until imaging exams were performed.

**Figure 1:**
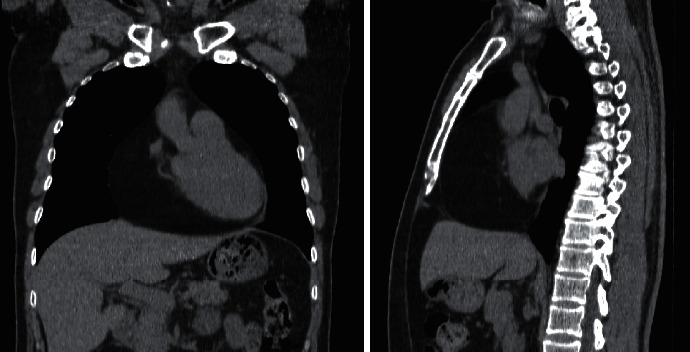
Voluminous pericardial mass. Pericardium tumor with a density similar to fat on a computed tomography scan. Note that the tumor causes posterior displacement of the heart and signs of a reduction in the dimensions of the right heart chambers.

Transesophageal echocardiography revealed a 79% ventricular ejection fraction and a hypoechoic mass within the pericardium attached to the free wall of the right ventricle and atria. A computed tomography scan showed an expansive mass in the anterior region of the pericardium with a homogeneously low density similar to fat. The tumor had a regular shape and measured 14.0 × 10.0 × 16.0 cm. The cardiac lesion extended from the upper to the lower thoracic region, displaced the heart backward, and reduced the right cardiac chambers (Figure 1).

**Figure 2:**
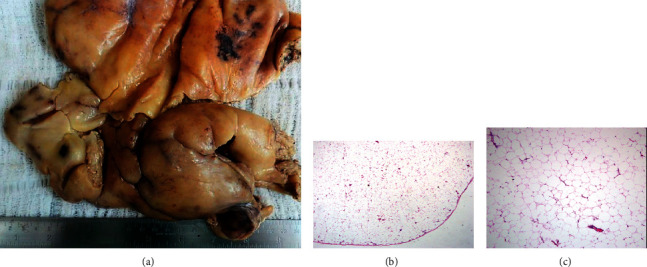
Voluminous intrapericardial lipoma. (a) Gross examination—fatty lobulated massive tumor covered by a fine fibrous capsule. (b) Microscopy—delicate fibrous capsule delineates the mass. (c) Mature adipocytes form the tumor. No atypia is observed.

The patient was submitted to a pericardial window via a subxiphoid surgical approach, in which a pedunculated epicardial tumor inserted in the right ventricle was resected. No cardiopulmonary bypass was needed. At gross examination, the tumor was split into two irregular fatty fragments measuring 22.5 × 9.0 × 4.0 cm and 21.0 × 15.0 × 2.0 cm and weighing 635 grams (Figure 2(a)). The histological exam revealed an encapsulated neoplasm formed by mature adipocytes (Figures 2(b) and 2(c)). No lipoblasts, cellular atypia, or mitosis were seen, and the diagnosis of lipoma was stated.

Ten days after surgery, the patient was discharged from the hospital without any medications. At a follow-up at 90 days, the patient was completely asymptomatic.

## 3. Discussion

Most cardiac lipomas are asymptomatic. Therefore, the diagnosis of these benign neoplasms is frequently done on autopsies. Medical literature may underestimate their real prevalence [18]. Generally, they remain indolent for many years since clinical symptoms depend on the growth speed, position, and tumor size [19].

The majority of cardiac lipomas is sessile or polypoid masses implanted in the subendocardium or epicardium. Only 25% of cardiac lipomas arise within the myocardium [11, 20]. Often, lipomas arise from the epicardial fat and grow into the pericardial sac [11]. However, there are case reports of distinct sites, such as the right atrium [10], left atrium [21, 22], tricuspid valve [23, 24], mitral valve [25], right ventricle [26], left ventricle [20, 27], interventricular septum [12, 28], and inside the ventricular cavities themselves [29]. Size ranges from 1 to 15 cm in diameter, and weight such as 2 kg is described [20].

Symptoms occur when the mass compresses the cardiac chambers and causes hemodynamic changes. Sometimes, it may cause pericardial effusions [30]. Intrapericardial lipomas can cause dyspnea by reducing the pulmonary volume or by elevating the ventricular filling pressures [31]. Fatigue and thoracic pain, possibly by coronary artery compression, are less common clinical manifestations [11, 17]. Intracavitary lipomas can lead to heart failure, syncope, and sudden death [19]. Intramyocardial lipomas may result in conduction system disorders and arrhythmias [4, 32–35]. Our patient presented thoracic pain and dyspnea due to a voluminous intrapericardial lipomatous tumor compressing the cardiac chambers.

Chest radiography, transthoracic and transesophageal echocardiogram, tomography scan, and magnetic resonance imaging play an important role in identifying the lipoma location and relationship to the heart [4, 5, 8, 9, 11, 36–38]. On chest radiography, cardiac lipomas may mimic hilar neoplasia, diaphragmatic herniation, or mediastinal mass [19]. An echocardiogram can usually define the position and size of the lipoma [11]. Tomography scan and magnetic resonance imaging establish the fatty differentiation of the tumor, define precisely its insertion and relationship to the cardiac structures, and give additional information to rule out malignant neoplasms, such as liposarcomas [4, 8, 11, 39]. Coronary angiography can be useful in cases of coronary compression [4, 19].

Imaging exams are important to evaluate the biological behavior of heart tumors. However, malignancy and the definitive diagnosis can be made by histopathological examination only [18]. On gross examination, lipomas are bright yellow fatty nodules with a delicate capsule. The cut surface is greasy with fine fibrous trabeculae. Under the microscope, lipomas are constituted by mature adipose tissue. There may be areas of steatonecrosis and calcification. Ruling out well-differentiated liposarcomas can be problematic in some cases. The latter contains a few lipoblasts [11]. In our case, the histological exam excluded malignant criteria.

Although lipomas are benign and slow-growing tumors, the surgical approach is to prevent heart compression and hemodynamic changes [4, 11]. Surgery has satisfactory results and low mortality [8, 11, 40]. The complete removal of the tumor pedicle is crucial to avoid relapses [8, 11, 16, 26, 41]. Surgery scheduling depends upon many factors, especially on the tumor size and location [42, 43]. Our patient underwent an elective surgery with no complications nor relapses at his follow-up.

Pericardial lipomas' signs and symptoms are variable. Indeed, most of the patients are asymptomatic. Conversely, surgical removal is necessary to prevent cardiac compression and to exclude malignancy through the histopathological examination.

## Data Availability

The medical data used to support the findings of this study are restricted in order to protect patient privacy.
